# Enhanced IFN-α production is associated with increased TLR7 retention in the lysosomes of palasmacytoid dendritic cells in systemic lupus erythematosus

**DOI:** 10.1186/s13075-017-1441-7

**Published:** 2017-10-19

**Authors:** Goh Murayama, Nanako Furusawa, Asako Chiba, Ken Yamaji, Naoto Tamura, Sachiko Miyake

**Affiliations:** 1 Department of Immunology, Juntendo University School of Medicine, 2-2-1 Hongo, Bunkyo-ku, Tokyo, 113-8421 Japan; 2 Department of Internal Medicine and Rheumatology, Juntendo University School of Medicine, Tokyo, Japan

**Keywords:** Systemic lupus erythematosus, Toll-like receptor, Interferon-α, Plasmacytoid dendritic cells

## Abstract

**Background:**

Interferon-α (IFN-α) is increased and plays an important role in the pathogenesis of systemic lupus erythematosus (SLE). Plasmacytoid dendritic cells (pDCs) are the main producer of IFN-α, but their IFN-α producing capacity has been shown to be unchanged or reduced when stimulated with a Toll-like receptor 9 (TLR9) agonist in patients with SLE compared to in healthy individuals. In this study, we investigated the IFN-α-producing capacity of lupus pDCs under different stimulation.

**Methods:**

pDCs from patients with SLE and healthy controls (HC) were stimulated with TLR9 or TLR7 agonist, and their IFN-α producing capacity was examined by intracellular cytokine staining and flow cytometry. The correlation of IFN-α-producing capacity with serum IFN-α levels and disease activity was assessed. The effect of in vitro IFN-α exposure on IFN-α production by pDCs was examined. Localization of TLR7 in cellular compartments in pDCs was investigated.

**Results:**

The IFN-α producing capacity of pDCs was reduced after TLR9 stimulation, but increased when stimulated with a TLR7 agonist in SLE compared to in HC. IFN-α production by pDCs upon TLR9 stimulation was reduced and the percentage of IFN-α^+^pDC was inversely correlated with disease activity and serum IFN-α levels. However, the TLR7 agonist-induced IFN-α producing capacity of lupus pDCs was enhanced and correlated with disease activity and serum IFN-α. Exposure to IFN-α enhanced IFN-α production of TLR7-stimulated pDCs, but reduced that of pDCs activated with a TLR9 agonist. TLR7 localization was increased in late endosome/lysosome compartments in pDCs from SLE patients.

**Conclusions:**

These findings indicate that enhanced TLR7 responses of lupus pDCs, owing to TLR7 retention in late endosome/lysosome and exposure to IFN-α, are associated with the pathogenesis of SLE.

**Electronic supplementary material:**

The online version of this article (doi:10.1186/s13075-017-1441-7) contains supplementary material, which is available to authorized users.

## Background

Type I interferons (IFNs) including IFN-α are known to play important roles in the pathogenesis of systemic lupus erythematosus (SLE) [1–3]. Over expression of type I IFNs has been shown to accelerate production of autoantibodies and tissue damage in lupus-prone mice [4]. Deletion of the type I IFN receptor results in reduced autoantibody production and disease severity [5, 6]. Treatment of patients with hepatitis C virus or cancer with type I IFN induces lupus-like disease [7–9]. Early studies showed that the levels of type I IFN or IFN-inducible genes in peripheral blood mononuclear cells (PBMCs) are increased in patients with SLE, and that the levels of IFN-α are associated with disease severity [10–19]. Genome-wide association studies have shown that molecules involved in the production of type I IFN affect SLE susceptibility [20–22]. Lupus-associated single-nucleotide polymorphisms are found in genes encoding molecules that participate in the production of IFN-α, such as Toll-like receptor (TLR) 7, IFN-regulatory factor (IRF) 5, and IRF7 [23–30]. Lupus-associated single-nucleotide polymorphisms in IRF7, IRF5, and STAT4 are related to elevated levels of IFN-α, and some SLE risk haplotypes of IRF5 have been shown to be associated with increased IFN-α expression in SLE [27, 31].

Plasmacytoid dendritic cells (pDCs) are the main producers of IFN-α [32–35]. IFN-α production by human pDCs occurs mostly through the TLR7 and TLR9 signaling pathways. Under physiological conditions, TLR7 recognizes single-strand RNA and TLR9 responds to DNA from viruses or bacteria. In SLE, immune complexes composed of autoantibodies and DNA or RNA have been shown to induce IFN-α production by pDCs [36–39]. Although many studies have demonstrated an increase in serum IFN-α levels or IFN-inducible genes in SLE, as to whether the IFN-α-producing capacity of pDCs is enhanced in SLE remains unclear. IFN-α production by CD123^+^ cells after stimulation with influenza virus is comparable between SLE patients and healthy donors [40]. Other reports show that IFN-α production by PBMCs or pDCs after stimulation with herpes simplex virus, Sendai virus, or CpG oligodeoxyribonucleotide (ODN) is reduced in patients with SLE [41–45]. These findings suggest that the IFN-α-producing capacity of lupus pDCs varies depending on the type of stimuli.

In the current study, we investigated the IFN-α-producing capacity by pDCs after stimulation with TLR7 or TLR9 agonists. IFN-α production by pDCs upon TLR9 stimulation was reduced, and the percentage of IFN-α^+^pDCs was inversely correlated with disease activity and serum IFN-α levels. However, the IFN-α-producing capacity of lupus pDCs was enhanced following stimulation with a TLR7 agonist and correlated with disease activity and serum IFN-α. This is the first study demonstrating enhanced IFN-α producing capacity of lupus pDCs. We also found that prior exposure to IFN-α enhanced the IFN-α producing capacity of pDCs after stimulation with a TLR7 agonist, but reduced TLR9 agonist-induced IFN-α production by pDCs. Finally, we demonstrate that enhanced IFN-α production by TLR7-stimulated pDCs was associated with increased retention of TLR7 in late endosome/lysosome compartments in lupus pDCs. Thus, the enhanced IFN-α-producing capacity of pDCs, owing to increased TLR7 in late endosome/lysosome compartments, is augmented by exposure to IFN-α during active disease.

## Methods

### Subjects

Our study with flow cytometric analysis included 71 patients diagnosed with SLE (66 women and 5 men, median age (interquartile range (IQR)) 36.0 years (22.0–60.0)) and 45 healthy controls (HC) (41 women and 4 men, median age 36.0 years (21.0–56.0)). The study with confocal microscopic analysis included six patients with SLE and five HCs (Additional file 1: Table S1). We obtained peripheral blood from patients with SLE and HC after obtaining informed consent in accordance with the local ethical committee guidelines of Juntendo University. SLE was diagnosed according to the American College of Rheumatology criteria for SLE. HC did not have a history of any autoimmune disease and had never received immunosuppressive therapy. Informed consent was obtained from all patients with SLE and all HC according to the ethical guidelines for human subject research. Disease activity was assessed by the SLE Disease Activity Index 2000 (SLEDAI-2 K) [46]. Active disease was defined as a SLEDAI-2 K score > 4. The ages, sex, and treatments of the patients are presented in Table 1.

**Table 1:** Characteristics of healthy controls (HC) and patients with systemic lupus erythematosus (SLE)

	HC	SLE
Number	45	71
Female/male, *n*	41/4	66/5
Age, years	36.0 (21.0–56.0)	36.0 (22.0–60.0)
Disease duration, years		7.0 (0.0–31.0)
Anti-DNA antibody (IU/mL)		3.5 (2.0–300.0)
C3 (mg/dL)		67.0 (9.0–195.0)
CH50 (U/mL)		29.4 (2.0–78.1)
SLEDAI score		4.0 (2.0–34.4)
SLEDAI < 5, *n*		41
SLEDAI ≥ 5, *n*		30
Disease		
Glomerulonephritis, n		37
Arthritis, *n*		11
Neuropsychiatric SLE, *n*		8
Pancytopenia, *n*		6
Lupus enteritis, *n*		5
Cutaneous lupus erythematosus, *n*		3
Pneumonitis, *n*		1
Medications		
Medication naïve, *n*		10
Prednisone, *n*		57
Prednisone dose (mg/day)		7.0 (2.0–45.0)
Immunosuppressive agent^a^, *n*		19

Values are number or median (interquartile range)

*SLEDAI* Systemic Lupus Erythematosus Disease Activity Index 2000

^a^Azathioprine, mizoribine, mycophenolate mofetil, tacrolimus

### Flow cytometry

Fresh PBMCs were isolated from whole blood by density-gradient centrifugation using the BD Vacutainer CPT Mononuclear Cell Preparation Tubes with Sodium Heparin (BD Biosciences, Franklin Lakes, NJ, USA). The cells were first stained using the Zombie Yellow™ Fixable Viability Kit (BioLegend, San Diego, CA, USA) and then with combinations of the following monoclonal antibodies against human cell-surface antigens for 30 min on ice: anti-CD11c-Alexa700, anti-HLADR-V500, anti-CD19-APC-H7 (all from BD Biosciences), anti-CD14-ECD, anti-CD56-APC, (both from Beckman Coulter, Brea, CA, USA), anti-CD123-FITC, anti-CD3- PerCPCy5.5, anti-CD56-BV421 (all from BioLegend), and anti-CD19-PE (TONBO Biosciences, San Diego, CA, USA). pDCs were identified as CD3^-^CD19^-^CD14^-^CD56^-^HLADR^+^CD11c^-^CD123^+^(Additional file 2: Figure S1). Data were acquired on a FACS LSR Fortessa (BD Biosciences) and the percentages of each cell population and mean fluorescence intensity were analyzed using FlowJo software (TreeStar Inc., Ashland, OR, USA).

### TLR stimulation and intracellular cytokine staining

PBMCs were cultured in 96-well flat-bottom plates in Basal Medium Eagle (Thermo Fisher Scientific, Waltham, MA, USA) supplemented with 10% fetal bovine serum, 2 mM L-glutamine, 50 U/mL penicillin, and 50 μg/mL streptomycin (all from Thermo Fisher Scientific). PBMCs were stimulated with recombinant Human IL-3 (100 ng/mL; PEPROTECH, Rocky Hill, NJ, USA) and a TLR7 agonist, imiquimod (R837) (100 ng/mL; InvivoGen, San Diego, CA, USA) or a TLR9 agonist, CpG ODN 2216 (5 μg/mL; Miltenyi Biotec, Bergisch Gladbach, Germany) for 6 h at 37 °C in a 5% CO_2_ incubator. GolgiPlug (100 ng/mL; BD Biosciences) was added during the final 3 h of stimulation to block cytokine secretion. After staining the cell-surface antigens, intracellular cytokines were stained using the BD Cytofix/Cytoperm Fixation/Permeabilization Solution Kit (BD Biosciences), anti-IFN-α-APC (Miltenyi Biotec), and anti-tumor necrosis factor α (TNF-α)-PE-Cy7 (BD Biosciences), or their isotype control antibodies.

### Pretreatment with cytokines

PBMCs were cultured in culture medium with IFN-α (100 U/mL) (R&D Systems, Minneapolis, MN, USA) for 24 h at 37 °C in 5% CO_2_. After pretreatment with IFN-α, cells were stimulated with TLR agonists, and intracellular cytokine staining was performed as described above.

### Measurement of serum IFN-α

Levels of serum IFN-α were determined in patients with SLE and in HC using VeriKine-HS Human Interferon Alpha All Subtype ELISA Kit (PBL Assay Science, Piscataway Township, NJ, USA) according to the manufacturer’s instructions.

### Confocal microscopy

DCs were purified from PBMCs from patients with SLE and from HC using a Pan-DC Enrichment Kit (Miltenyi Biotec, Bergisch Gladbach, Germany) according to the manufacturer’s instructions. Purified DCs were spun onto a microscope slide using the Thermo Shandon Cytospin 4 (Thermo Fisher scientific, MA, USA). DCs were fixed with 4% paraformaldehyde and then permeabilized with Triton X-100 (0.2% Triton X-100 in PBS). Nonspecific background staining was prevented by incubating with Image-iT FX Signal Enhancer (Thermo Fisher scientific, MA, USA). Cells were incubated for 1.5 h at room temperature with primary antibodies: anti-TLR7 (Novus Biologicals, CO, USA), anti-BDCA2 (Novus Biologicals), anti-KDEL, anti-Early Endosome Antigen1 (EEA1), anti Rab7 and anti-lysosomal associated membrane protein-1 (LAMP1) (all from Abcam, MA, USA), and then washed and incubated for 1.5 h at room temperature with secondary antibodies: Alexa488-donkey anti-mouse IgG, Alexa594-donkey anti-goat IgG and Alexa647-donkey anti-rabbit IgG ( all from Jackson ImmunoResearch Laboratories, PA, USA). Cell nuclei were stained with 4’,6-diamidino-2-phenylindole (DAPI) (Sigma Aldrich, MO, USA) and mounted with Fluoromount/Plus (Diagnostic BioSystems, CA, USA). All samples were visualized using the FM1000D confocal laser scanning microscope (Olympus, Tokyo, Japan), and images were captured and analyzed using the FV10-ASW viewer (Olympus). pDCs were identified as BDCA2-positive cells. Pearson’s correlation was calculated using ImageJ, for quantitative analysis of the co-localization of TLR7 and endosomal markers (KDEL, EEA1, Rab7 and LAMP1).

### Statistical analysis

All data were analyzed using GraphPad Prism (GraphPad, Inc., La Jolla, CA, USA) and differences between groups were analyzed using the Mann-Whitney *U* test or Kruskal-Wallis test followed by Dunn’s multiple comparisons test. The significance level was set at *p* < 0.05. Associations between two variables were analyzed using Spearman correlation.

## Results

### IFN-α production by pDCs upon TLR9 stimulation was reduced and inversely correlated with disease activity in SLE

**Fig. 1:**
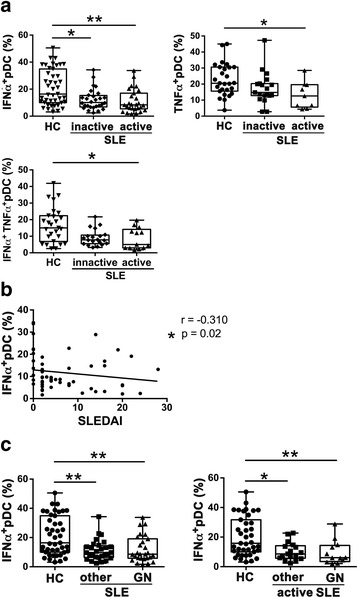
Toll-like receptor (TLR)9-induced interferon-α (IFN-α) production by plasmacytoid dendritic cells (pDCs) was reduced and was inversely correlated with disease activity in systemic lupus erythematosus (SLE). Peripheral blood mononuclear cells (PBMCs) were stimulated with CpG ODN and cytokine producing pDCs were analyzed by flow cytometry in healthy controls (HC) and patients with SLE with inactive or active disease. **a** The proportions of pDCs producing IFN-α, TNF-α, or both IFN-α and TNF-α are shown. Each symbol represents the value of one individual. The box plot indicates the first and third quartiles and the middle line indicates the median. Whiskers indicate the minimum and maximum. *p < 0.05, **p < 0.01 (Kruskal-Wallis test followed by Dunn’s multiple comparisons test). **b** The correlation of the frequency of CpG-induced IFN-α-producing pDCs with SLE activity (SLE Disease Activity Index 2000 (SLEDAI)) score was examined using Spearman correlation. Each symbol represents the value of one individual. **c** Frequencies of CpG-induced IFN-α-producing pDCs were compared between patients with glomerulonephritis (GN) or other manifestations, in all patients (*left*) or in patients with active disease (*right*)

We first investigated IFN-α production by pDCs stimulated with a TLR9 ligand, CpG ODN2216 (hereafter referred to as CpG) in patients with SLE and HC. As previously reported by other groups [42–44], the percentages of IFN-α-producing cells among pDCs were reduced in patients with SLE. Although there was no statistical difference in the percentages of IFN-α^+^ pDCs between patients with inactive and active SLE, there was greater reduction in the percentage of IFN-α^+^ pDCs in patients with active disease compared to in HC (Fig. 1a). pDCs also produced TNF-α when stimulated with CpG. TNF-α production by pDCs was decreased in patients with active disease, but not inactive disease, compared to HC. The percentage of IFN-α^+^TNF-α^+^ pDCs was also decreased in patients with active disease. The IFN-α producing capacity by pDCs appears to be related to disease activity. The frequency of IFN-α^+^ pDCs was negatively correlated with the SLEDAI (Fig. 1b). We also assessed whether the reduced IFN-α producing capacity of pDCs was related to the involvement of clinical manifestations. The percentages of IFN-α^+^ pDC were reduced in all groups of patients with or without active glomerulonephritis or other clinical phenotypes (Fig. 1c).

### IFN-α production by pDCs stimulated with a TLR7 agonist was increased and positively correlated with disease activity in SLE

**Fig. 2:**
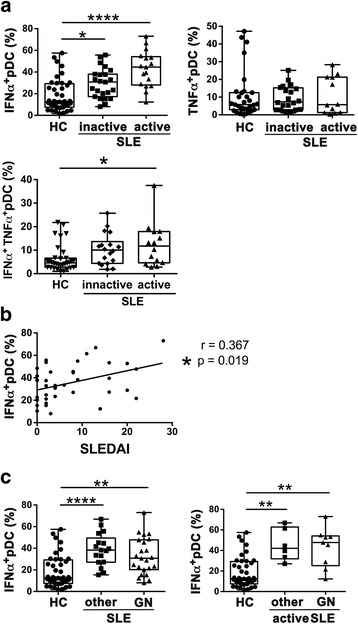
Toll-like receptor 7 (TLR7)-induced interferon- α (IFN-α) production by plasmacytoid dendritic cells (pDCs) increased and was positively correlated with disease activity in systemic lupus erythematosus (SLE). Peripheral blood mononuclear cells (PBMCs) were stimulated with imiquimod and cytokine-producing pDCs were analyzed by flow cytometry in healthy controls (HC) and patients with SLE with inactive or active disease. **a** The proportions of pDCs producing IFN-α, TNF-α, or both IFN-α and TNF-α are shown. Each symbol represents the value of one individual. The box plot indicates the first and third quartiles and the middle line indicates the median. Whiskers indicate the minimum and maximum. *p < 0.05, **p < 0.01, ****p < 0.001 (Kruskal-Wallis test followed by Dunn’s multiple comparisons test). **b** The correlation of the frequency of imiquimod-induced INF-α-producing pDCs with SLE activity (SLE Disease Activity Index 2000 (SLEDAI)) score was examined using Spearman correlation. Each symbol represents the value of one individual. **c** Frequencies of imiquimod-induced IFN-α-producing pDCs were compared between patients with glomerulonephritis (GN) or other manifestations, in all patients (*left*) or in patients with active disease (*right*))

Next, we evaluated whether IFN-α production by lupus pDCs was reduced following activation by other TLR pathways. Surprisingly, as shown in Fig. 2a, IFN-α production by pDCs was enhanced in SLE when stimulated with a TLR7 ligand, imiquimod, and there was a greater increase in the percentage of IFN-α^+^ pDCs in patients with SLE with active disease compared to those with inactive disease. The percentage of IFN-α^+^TNF-α^+^ pDCs was higher in patients with active disease. In addition, the percentage of IFN-α^+^ pDCs was positively correlated with the SLEDAI (Fig. 2b). Although imiquimod stimulation induced TNF-α production by pDCs, there was no difference between HC and patients with SLE (Fig. 2a). The percentages of IFN-α^+^ pDCs were higher in both patient groups with or without glomerulonephritis than in HC (Fig. 2c).

### Increased IFN-α production by pDCs was not associated with treatments in SLE

**Fig. 3:**
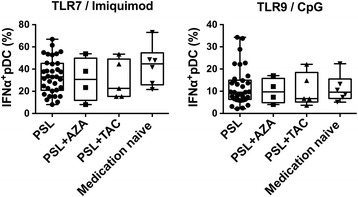
Increased interferon-α (IFN-α) production by plasmacytoid dendritic cells (pDCs) was not associated with treatments in systemic lupus erythematosus (SLE). The proportions of pDCs producing IFN-α in patient groups receiving different medications from the data shown in Figs. 1 and 2; patient samples examined in both experiments are included. Each symbol represents the value of one individual. The box plot indicates the first and third quartiles and the middle line indicates the median. Whiskers indicate the minimum and maximum. *p < 0.05, **p < 0.01, ****p < 0.001 (Kruskal-Wallis test followed by Dunn’s multiple comparisons test). PSL prednisone, AZA azathioprine, TAC tacrolimus

Immunosuppressive agents, including corticosteroids, could have an effect on the cytokine producing-capacity of pDCs. To investigate whether the medication affected the IFN-α production of pDCs in SLE, we compared the percentage of IFN-α^+^ pDCs stimulated with CpG or imiquimod. There was a slight increase in the percentage of IFN-α^+^ pDCs when stimulated with imiquimod among patients that had not received treatment, but there was no statistically significant difference in the percentage of IFN-α^+^ pDCs among patients under different treatment regimens (Fig. 3). The percentage of IFN-α^+^ pDCs stimulated with CpG was also comparable among patient groups (Fig. 3).

### Serum IFN-α level positively correlates with IFN-α producing capacity of imiquimod-stimulated pDCs in SLE

**Fig. 4:**
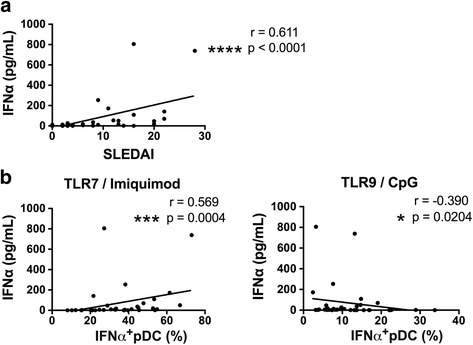
Serum interferon- α (IFN-α) level is positively correlated with IFN-α producing capacity of imiquimod-stimulated plasmacytoid dendritic cells (pDCs) in systemic lupus erythematosus (SLE). Serum levels of IFN-α were measured by ELISA. The correlation of the serum IFN-α level with the SLE Disease Activity Index 2000 (SLEDAI) (**a**) and frequencies of CpG or imiquimod-induced IFN-α-producing pDCs (**b**) were examined by Spearman correlation

IFN-α is known to be increased in the serum of patients with SLE. As previously reported, serum levels of IFN-α were increased in patients with SLE and were positively correlated with the SLEDAI (Fig. 4a). Thus, we next investigated whether IFN-α production by pDCs is related to the serum levels of IFN-α. There was positive correlation between the serum level of IFN-α and frequency of IFN-α^+^ pDCs stimulated with imiquimod (Fig. 4b). We also found that serum IFN-α levels were negatively correlated with the percentage of IFN-α^+^ pDCs stimulated with CpG (Fig. 4b). These results suggest that elevated serum IFN-α is associated with the IFN-α producing capacity of lupus pDCs.

### IFN-α enhanced IFN-α production of imiquimod-stimulated pDCs and suppressed IFN-α production of CpG-stimulated pDCs

**Fig. 5:**
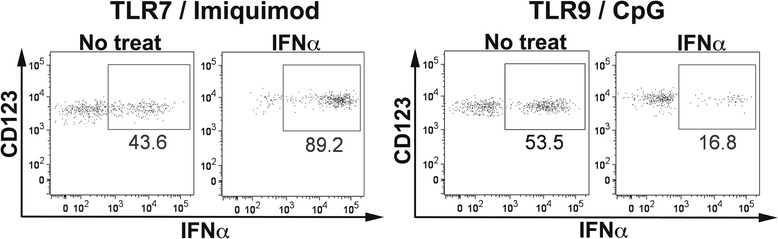
Interferon-α (IFN-α) enhanced imiquimod-induced IFN-α production and suppressed CpG-induced IFN-α production of plasmacytoid dendritic cells (pDCs). Imiquimod or CpG-induced IFN-α-producing capacity of pDCs pretreated with IFN-α was evaluated by intracellular cytokine staining. Values in graphs indicate percentages of IFN-α^+^ pDCs. Representative data of the IFN-α staining profile of pDCs from three independent experiments are shown. No treat = no treatment

To examine whether elevated levels of IFN-α influence IFN-α production by pDCs in SLE, PBMCs from healthy volunteers were treated with IFN-α for 24 h and then IFN-α production by pDCs was measured after stimulation with CpG or imiquimod. Culture in medium without cytokines for 24 h reduced IFN-α production by pDCs upon stimulation with either imiquimod or CpG. Pretreatment with IFN-α enhanced IFN-α production by pDCs stimulated with imiquimod, but reduced CpG-stimulated IFN-α production of pDCs (Fig. 5).

### IFN-α production was observed only in stimulated pDCs

**Fig. 6:**
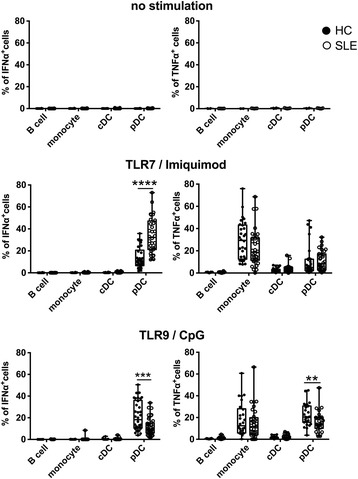
Inteferon-α (IFN-α) production was observed only in stimulated plasmacytoid dendritic cells (pDCs). The proportions of IFN-α-producing or TNFα-producing cells unstimulated or stimulated with imiquimod or CpG from experiments in Figs. 1 and 2 are shown. Data are shown as median + standard deviation. **p < 0.01, ***p < 0.0005 ****p < 0.001 (Mann-Whitney test). cDC conventional DCs

We next evaluated spontaneous IFN-α production in pDCs and other immune cell subsets, including B cells, monocytes and conventional DCs (cDCs) and whether this might affect the cytokine-producing capacity of pDCs. Without stimulation, no cells produced IFN-α or TNF-α (Fig. 6). We also investigated IFN-α or TNF-α production by these cell subsets upon stimulation with CpG or imiquimod. Monocytes and cDCs produced TNF-α upon stimulation, but none of the cells except pDCs produced IFN-α (Fig. 6).

### TLR7 localization was increased in late endsomal and lysosomeal compartments in pDCs from patients with SLE

**Fig. 7:**
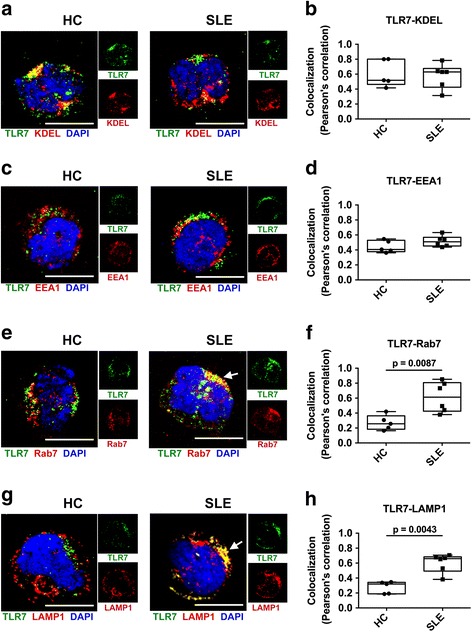
Enhanced localization of Toll-like receptor 7 (TLR7) in late endosomal and lysosomal compartments in lupus plasmacytoid dendritic cells (pDCs). (**a**-**h**) DCs from healthy controls (HC) or patients with systemic lupus erythematosus (SLE) stained with antibodies to BDCA2, TLR7, KDEL and EEA1, Rab7, LAMP1 and DAPI (blue). Representative images of BDCA2-positive pDCs from HC and pPatients with SLE stained with TLR7 (*green*) and indicated markers (*red*) of endocytic pathways are shown (**a, c**, **e**, **g**). Scale bars indicate 5.0 μm and *white arrows* indicate robust co-localization of TLR7 with Rab7 and LAMP1. Quantificaion of co-localization between TLR7 and KDEL (**b**), EEA1 (**d**), Rab7 (**f**) and LAMP1 (**h**) is shown. Each symbol represents the value of one individual. The *box plot* indicates the first and third quartiles and the middle line indicates the median. Whiskers indicate the minimum and maximum. The Mann-Whitney test was used to test for the differences between pDCs from HC and patients with SLE

It is known that the TLR7 and TLR9 signaling pathways are regulated by their localization in the ensosomal compartments and IFN-α production requires TLR trafficking to lysosome-related organelle [47]. To understand why IFN-α production of pDC stimulated with a TLR7 agonist was increased in SLE, we investigated the localization of TLR7 in pDC from HC and patients with SLE. Co-localization of TLR7 with KDEL (endoplasmic reticulum marker) or EEA1 (early endosome marker) was comparable between pDCs from HC and patients with SLE (Fig. 7). On the other hand, co-localization between TLR7 and Rab7 (late endosome marker) or LAMP1 (lysosome marker) was increased in pDCs from patients with SLE compared to those from HC (Fig. 7). In pDCs from two patients with SLE with active disease, co-localization of TLR7 seemed to be more increased with LAMP1 than KDEL. The Pearson’s correlation coefficient of TLR7 and LAMP1 was 0.706 and 0.668, whereas that of TLR7 and KDEL was 0.314 and 0.462. These results indicate that increased localization of TLR7 in lysosome may also be related to augmented IFN-α production of lupus pDCs stimulated with a TLR7 agonist.

## Discussion

Despite the increased levels of IFN-α in patients with SLE, previous studies showed either decreased or comparable IFN-α-producing capacity of lupus pDCs compared to healthy controls [40–45]. As previously reported, lupus pDCs produced lower levels of IFN-α after stimulation with a TLR9 agonist. However, the proportion of IFN-α-producing pDCs was greatly increased in patients with SLE when pDCs were activated with a TLR7 agonist. We also showed that the IFN-α-producing capacity of pDCs was associated with disease activity and serum IFN-α levels. We showed that exposure to IFN-α enhanced IFN-α production upon TLR7 stimulation, but reduced TLR9-induced IFN-α production. We further demonstrated that more TLR7 localized in late endosome and lysosome in pDCs from patients with SLE. These findings suggest that the enhanced IFN-α-producing capacity of pDCs resulting from increased TLR7 signaling was further augmented by exposure to IFN-α in SLE. Because animal studies showed that TLR7 plays an important role in disease progression [48–51], enhanced IFN-α production by pDCs may be associated with the pathogenesis of SLE. In this study, we tested the responses of pDCs against an artificial TLR7 ligand, imiquimod. It would be more informative to examine the responses of lupus pDCs against more pathophysiological ligands such as single-stranded RNA or RNA-IgG immune complexes.

The SLEDAI was positively correlated with both serum levels of IFN-α and frequencies of IFN-α^+^ pDCs stimulated with a TLR7 agonist. As IFN-α activates pDCs through the interferon receptor [52, 53], we evaluated whether IFN-α exposure enhances IFN-α production of pDCs. As expected, after in vitro treatment with IFN-α, the frequency of IFN-α^+^ pDCs stimulated with a TLR7 agonist was increased and TLR9-induced IFN-α production by pDCs was reduced. Because serum IFN-α is associated with the SLEDAI, the increased IFN-α^+^ production by pDCs after stimulation with a TLR7 agonist in patients with active disease may be due to prior exposure to IFN-α in vivo. Among other immune cell types tested, IFN-α production was only observed in pDCs. Thus, IFN-α production by stimulated pDCs could be modified by the IFN-α produced by themselves. Other cells, including monocytes and cDCs responded to TLR7 or TLR9 stimulation. Therefore, IFN-α production by pDCs could be affected by other cells activated by a TLR agonist through different mechanisms.

The decreased production of IFN-α by lupus pDCs upon TLR9 activation has been reported by other groups [43–45]. Prior exposure of pDCs to either a TLR9 agonist or lupus serum reduces IFN-α production by these cells. Because the levels of immune complexes in lupus sera are inversely correlated with CpG-induced IFN-α production in lupus PBMCs, pDCs activated in vivo, most likely through TLR9 stimulation with IC containing DNA, may become tolerant to further stimulation [44]. In our study, reduced CpG-induced IFN-α production by pDCs was negatively correlated with the SLEDAI. Therefore, such pDCs stimulated with IC in vivo may show a low response to further in vitro stimulation. However, pretreatment of pDCs with IFN-α reduced CpG-induced IFN-α production of these cells. In addition, the frequency of CpG-induced IFN-α^+^ pDCs was negatively correlated with the levels of serum IFN-α. This supports that the reduction of CpG-induced IFN-α^+^ pDCs is associated with increased IFN-α levels in SLE.

IFN-β has been demonstrated to upregulate TLR7 in pDCs, but not in other cell subsets including monocytes, myeloid DCs, B cells, and T cells [54]. In vivo treatment with IFN-β induces TLR7 upregulation and TLR9 down modulation in PBMCs of patients with multiple sclerosis [54]. IFN-α may have similar effects on TLR expression by pDCs in SLE. Further studies on the effect of IFN-α on the expression of TLRs may be important to understand the IFN-α-producing capacity of lupus pDCs.

TLR7 and TLR9 share downstream signaling pathways [55]. The first pathway requiring nuclear factor κB activation leads to production of proinflammatory cytokines such as IL-12 and TNF-α. The second pathway leads to the IRF7-dependent production of type I IFN. TNF-α production by pDCs was comparable between HC and patients with SLE both after TLR7 and TLR9 stimulation. However, although lupus pDCs responded strongly to imiquimod stimulation and produced higher levels of IFN-α, they produced decreased levels of IFN-α when stimulated with CpG. This second pathway requires trafficking of TLRs from early endosome to lysosome-related organelle. As we found increased TLR7 co-localization with Rab7 and LAMP1 in pDCs from patients with SLE, the IRF signaling pathway appeared to be more active in lupus pDCs. As good antibodies against TLR9 were not commercially available, we were not able to investigate the location of TLR9 in cellular compartments. Thus, why lupus pDCs responded in an opposite manner in terms of IFN-α production after stimulation of similar signaling pathways requires further analysis. Uncoordinated 93 homolog B1 (UNC93B1) is an important molecule for endosomal TLR trafficking, and UNC93B1 discriminates between TLR7 and TLR9 [56]. Therefore, TLR chaperones and trafficking factors may be related to the IFN-α-producing capacity of lupus pDCs. It is also not known how exposure to IFN-α regulates the TLR7/9 downstream pathway or which other mechanisms are involved in IFN-α production by pDCs in SLE. TLR9 has been suggested to be the main receptor for IC-containing DNA in SLE. More recent studies indicated that DNA binds to other nucleic acid receptors present in the cytoplasm [57]. Therefore, the responses of pDCs to other nucleic acid receptors in SLE remain unclear.

In murine lupus models, the role of TLR9 in the pathogenesis is unclear. TLR9 has been suggested to be required for the generation of autoantibodies against DNA [58], but TLR9 deficiency results in increased IFN-α and antibody levels, and disease exacerbation [48, 59, 60]. In contrast, several reports indicate that TLR7 is involved in the progression of autoimmune responses [48–51]. Deletion of TLR7 in MRL/lpr mice has been shown to reduce antibody production and nephritis [48]. Mice bearing the Y chromosome-linked genomic modifier *Yaa*, overexpress TLR7, which promotes autoreactive and inflammatory responses [50, 51]. Together with the results of human genetic studies identifying TLR7 and its signaling molecules as susceptible genes, the TLR7 pathway appears to play an important role in the pathogenesis of SLE.

## Conclusions

In the present study, we demonstrated that the enhanced IFN-α production by pDCs stimulated with a TLR7 agonist was associated with increased TLR7 location in late endosome and lysosome compartments in pDCs from patients with SLE. The importance of the TLR7 pathway and the role of pDCs in lupus pathology have been suggested in animal studies and human genetic studies [61, 62]. We observed enhanced TLR7 responses, further indicating involvement of the TLR7 pathway in the pathogenesis of SLE.

## Additional files

Additional file 1: Table S1. Characteristics of HC and lupus patients for confocal microscopic analysis. Values are n or median [interquartile range]. (DOCX 30 kb)

Additional file 2: Figure S1. Gating strategy of pDCs is shown. pDCs were identified as Zombie dye^-^ CD3^-^CD19^-^CD14^-^CD56^-^HLADR^+^CD11c^-^CD123^+^. (TIF 2794 kb)

## Abbreviations

CpG ODN: CpG oligodeoxyribonucleotide
ELISA: Enzyme-linked immunosorbent assay
HC: Healthy controls
IFN: Interferon
IL: Interleukin
PBMC: Peripheral blood mononuclear cell
PBS: Phosphate-buffered saline
pDC: Plasmacytoid dendritic cells
SLE: Systemic lupus erythematosus
SLEDAI: Systemic Lupus Erythematosus disease activity index
TLR: Toll-like receptor
TNF: Tumor necrosis factor
