# Supplementary material for: Enhanced IFN-α production is associated with increased TLR7 retention in the lysosomes of palasmacytoid dendritic cells in systemic lupus erythematosus
Source: Arthritis Res Ther. 2017 Oct 19;19:234. doi: 10.1186/s13075-017-1441-7 (PMC5649081; doi:10.1186/s13075-017-1441-7)
Supplement: Supplementary file 1 — Characteristics of HC and lupus patients for confocal microscopic analysis. Values are n or median [interquartile range]. (DOCX 30 kb) [file 13075_2017_1441_MOESM1_ESM.docx]

**Table S1**

Characteristics of HC and lupus patients for confocal microscopic analysis. Values are n or median [interquartile range].

HC SLE

Number 5 6

Females/males, n 5 6

Age, years 34.0[22.0, 41.0] 33.5[27.0, 45.0]

Disease duration, years 6.0[4.0, 15.0]

Anti-DNA antibody (IU/mL) 3.05[2.0, 30.0]

C3 (mg/dL) 73.5[35.0, 79.0]

CH50 (/mL) 38.1[11.4, 42.1]

SLEDAI score 2.0[0.0, 14.0]

SLEDAI < 5, n 4

SLEDAI ≥ 5, n 2

Disease

Glomerulonephritis, n 4

Arthritis, n 2

Medications

Medication naïve, n 0

Prednisone, n 6

Prednisone dose, mean ± SD (mg/day) 5.5[1.0, 10.0] Immunosuppressive agent*, n 2

* Azathioprine, Mycophenolate mofetil
