# Supplementary figures and images for: Enhanced IFN-α production is associated with increased TLR7 retention in the lysosomes of palasmacytoid dendritic cells in systemic lupus erythematosus
Source: Arthritis Res Ther. 2017 Oct 19;19:234. doi: 10.1186/s13075-017-1441-7 (PMC5649081; doi:10.1186/s13075-017-1441-7)

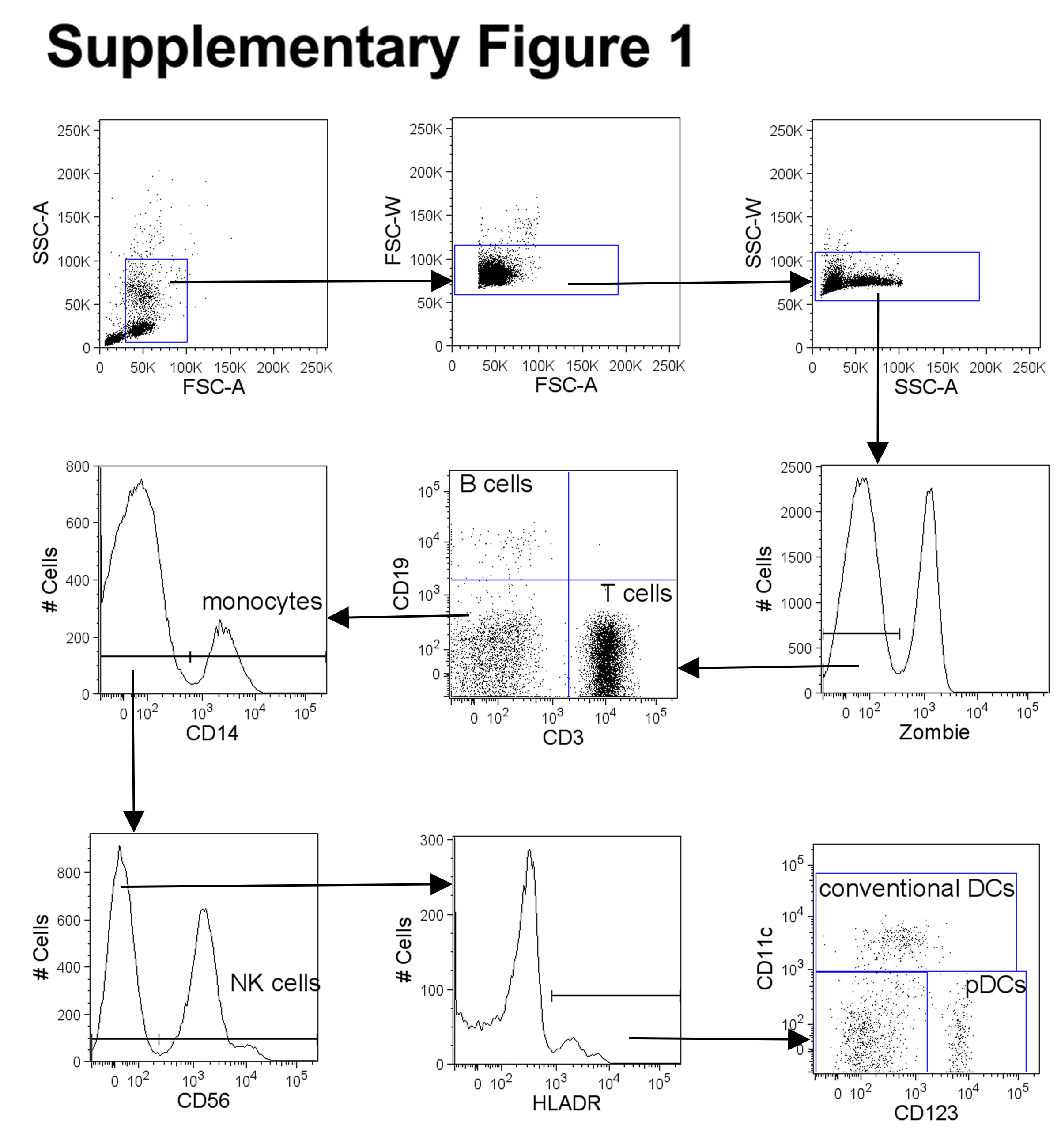

Supplement: Supplementary file 2 — Gating strategy of pDCs is shown. pDCs were identified as Zombie dye- CD3-CD19-CD14-CD56-HLADR+CD11c-CD123+. (TIF 2794 kb) [file 13075_2017_1441_MOESM2_ESM.tif]
